# Health-Related Quality of Life in Patients With Fibrosing Interstitial Lung Diseases: A Study Using the King’s Brief Interstitial Lung Disease (K-BILD) Questionnaire

**DOI:** 10.7759/cureus.117190

**Published:** 2026-09-30

**Authors:** Mamadou Siradjo Diallo, David Cherif, Gassima Brasseins, Jamal Oujaber, Amine Harzimi Benjelloun

**Affiliations:** 1 Department of Pulmonology, Faculté de Médecine et de Pharmacie, Université Cadi Ayyad, Marrakech, MAR; 2 Department of Pulmonology, Faculté des Sciences et Techniques de la Santé, Gamal Abdel Nasser University of Conakry, Conakry, GIN; 3 Department of Health, Faculté des Sciences et Techniques de la Santé, Gamal Abdel Nasser University of Conakry, Conakry, GIN; 4 Department of Pulmonology, Avicenne Military Hospital, Marrakech, MAR

**Keywords:** dyspnea, fibrosing interstitial lung disease, health-related quality of life, interstitial lung diseases, k-bild, oxygen saturation

## Abstract

Background and aim: Fibrosing interstitial lung diseases (ILDs) encompass a heterogeneous group of chronic, progressive pulmonary disorders characterized by varying degrees of pulmonary inflammation, fibrosis, and hypoxemia, which may ultimately lead to respiratory failure and death. Health-related quality of life (HRQoL) is an important outcome in patients with ILD. The primary objective was to assess HRQoL in patients with fibrosing interstitial lung diseases. The secondary objective was to explore the associations between K-BILD scores and selected sociodemographic, clinical, etiological, and treatment-related characteristics.

Methods: We conducted a cross-sectional observational study from January to July 2026 in the Department of Pulmonology at Avicenne Military Hospital in Marrakech, Morocco. We included patients with fibrosing ILD confirmed by chest computed tomography (CT). Sociodemographic, clinical, therapeutic, and etiological data were collected from medical records. Health-related quality of life (HRQoL) was assessed using a validated questionnaire.

Results: A total of 50 patients with fibrosing ILD were included. Males were predominant (56%), with a male-to-female ratio of 1.2. Patients' ages ranged from 50 to 86 years, with a mean age of 68 years. The 60-69-year age group was the most represented (46%). The mean total K-BILD score was 48.43±19.77, indicating an overall impairment in health-related quality of life in the study population. The breathlessness and activities domain had the lowest mean score (32.83±27.32), whereas higher mean scores were observed for the psychological domain (53.90±18.65) and the chest symptoms domain (56.44±22.76). Item-level analysis showed that item 1, which assesses breathlessness, had the lowest mean score (23.66±1.62). We observed a statistically significant association between the mean total K-BILD score and oxygen saturation on room air (p=0.005).

Conclusion: In our population of patients with fibrosing interstitial lung diseases (ILDs), health-related quality of life was overall impaired, with a mean total K-BILD score of 48.43±19.77. These findings highlight the potential value of systematic assessment of health-related quality of life as part of comprehensive management of patients with fibrosing ILDs. Further multicenter longitudinal studies involving larger patient populations and incorporating pulmonary function testing are warranted to confirm these associations.

## Introduction

Interstitial lung diseases (ILDs) refer to a broad group of pulmonary disorders characterized by inflammation and abnormal scarring of the pulmonary interstitium [1]. Diffuse infiltrative lung diseases encompass a wide variety of chronic and progressive pulmonary conditions characterized by varying degrees of pulmonary inflammation, fibrosis, and hypoxemia, which may ultimately lead to respiratory failure and death [2]. Pulmonary fibrosis is characterized by excessive and abnormal accumulation of extracellular matrix produced by activated fibroblasts and myofibroblasts in response to cytokines and growth factors, resulting in progressive remodeling and destruction of the normal tissue architecture of the affected organ [3]. According to the World Health Organization (WHO), quality of life is defined as an individual's perception of their position in life within the context of the culture and value systems in which they live, and in relation to their goals, expectations, standards, and concerns [4]. Pulmonary fibrosis is a chronic and disabling disease with a poor medium- and long-term prognosis, resulting in substantial functional impairment and deterioration in patients' quality of life [5]. In France, a study conducted in 2021 at Toulouse University Hospital and eight private healthcare institutions identified 256 cases of fibrosing ILD, corresponding to a minimum prevalence of 22.8 per 100,000 inhabitants and an estimated prevalence of 30.1 per 100,000. Idiopathic ILDs accounted for 55.8% of fibrosing ILDs, followed by ILDs associated with systemic diseases (24.6%) and exposure-related ILDs (11.3%). Idiopathic pulmonary fibrosis accounted for 35.9% of fibrosing ILDs, corresponding to a minimum prevalence of 8.2 per 100,000 and an estimated prevalence of 11.2 per 100,000 [6]. In a study conducted in a pulmonology department in Tunisia, an etiological investigation found that idiopathic pulmonary fibrosis accounted for 37.8% of cases [7]. At the Department of Pulmonology of Mohammed VI University Hospital in Marrakech, a study conducted in 2021 showed that the etiology of chronic fibrosing ILD was established in 32 patients (71.7%), including 15 cases (33.3%) of secondary ILD and 17 cases (37.8%) of idiopathic ILD, while the etiology remained undetermined in 13 patients (28.9%) [8]. Pulmonary fibrosis is a chronic and disabling disease with a poor medium- and long-term prognosis, resulting in substantial functional impairment and deterioration in patients' quality of life. Therefore, we aimed to assess the impact of pulmonary fibrosis on patients' daily activities and its effect on quality of life [5]. Early diagnosis and appropriate management are therefore crucial, as slowing disease progression in patients with fibrosing ILD may contribute to improving both quality of life and life expectancy [1]. The primary objective was to assess health-related quality of life (HRQoL) in patients with fibrotic interstitial lung diseases using the French version of the King’s Brief Interstitial Lung Disease (K-BILD) questionnaire. The secondary objective was to explore the associations between K-BILD scores and selected sociodemographic, clinical, etiological, and treatment-related characteristics.

## Materials and methods

Study design and setting

This was a single-center, cross-sectional observational study conducted in the Department of Pulmonology at Avicenne Military Hospital in Marrakech, Morocco. The study was conducted over seven months, from January 2026 to July 2026.

Study population and eligibility criteria

The study population consisted of patients managed in the Department of Pulmonology at Avicenne Military Hospital during the study period. Patients were eligible if they were followed in the department between January and July 2026 and had a documented diagnosis of fibrosing interstitial lung disease, established according to predefined clinical, biological, and radiological criteria. Patients were excluded if they declined to participate, had an unconfirmed diagnosis of pulmonary fibrosis, had an infectious pulmonary disease, or had a non-fibrosing interstitial lung disease.

Sampling technique and sample size

Exhaustive sampling was used. All patients with fibrosing ILD who attended the Department of Pulmonology during the study period and met the predefined eligibility criteria were considered for inclusion. We did not perform an a priori sample size calculation. Given the exhaustive nature of this single-center observational study over a predefined seven-month period, we included all eligible patients managed during the study period. In total, 50 patients met the eligibility criteria and were included in the final analysis. This approach was considered appropriate given the exploratory and single-center nature of the study.

Data collection

Data were collected during outpatient consultations using a pre-established data collection form. All participants underwent a clinical examination. Sociodemographic, clinical, etiological, and therapeutic data were collected from the patients and their medical records. The variables collected included age, sex, educational level, socioeconomic status, place of residence, marital status, health insurance coverage, personal and family medical history, disease onset and course, respiratory symptoms, clinical examination findings, disease etiology, and treatment modalities. For participants who were unable to read or write, the French version of the K-BILD questionnaire was administered by trained investigators who read each question and each response option verbatim and recorded the participant’s selected response without influencing or guiding their answers.

Study variables and assessment of health-related quality of life

Sociodemographic Data

Age, sex, educational level, socioeconomic status, place of residence, marital status, health insurance coverage, and financial independence, defined according to whether the patient required external assistance to meet basic needs or not.

Clinical Data

Data collected included personal and family medical history; smoking status (current smoker, former smoker, or never-smoker); disease onset (acute or progressive) and course; respiratory symptoms, including dyspnea assessed using the modified Medical Research Council (mMRC) scale (grades 1-4), cough, chest pain, and impaired general condition; clinical examination findings, including resting room-air oxygen saturation (SpO₂), measured by pulse oximetry after a standardized period of rest, and crackles on chest auscultation; disease etiology, based on the final etiological diagnosis established following multidisciplinary discussion; and treatment modalities, categorized as treatment received, specific medication administered, or no treatment.

Written permission to use the K-BILD questionnaire was obtained from Dr. Surinder S. Birring, one of the original developers of the questionnaire, prior to its use in this study. The primary outcome was health-related quality of life, assessed using the total K-BILD score. Secondary outcomes included the scores of the following three K-BILD domains: breathlessness and activities, psychological, and chest symptoms. The K-BILD questionnaire is a validated, disease-specific instrument for assessing health-related quality of life in patients with interstitial lung diseases. The original K-BILD consists of 15 items covering the following three domains: breathlessness and activities, psychological, and chest symptoms, with an additional item assessing the financial impact of the disease [9,10]. The French version used in this study was previously translated and linguistically and psychometrically validated [10]. The King's Brief Interstitial Lung Disease (K-BILD) questionnaire assesses patients' health status during the preceding two weeks and consists of 15 items (table in appendix).

Domain scores and the total score are transformed to a 0-100 scale, with higher scores indicating better health status and better health-related quality of life [10]. Item 15, which assesses the financial impact of the disease, does not belong to any of the three K-BILD domains. It was therefore analyzed separately and was not included in the calculation of either the domain scores or the total K-BILD score.

Potential Confounding Factors

Potential confounding factors included age, sex, smoking status, disease etiology, disease severity, degree of dyspnea, resting oxygen saturation, and treatment modalities. These variables were considered because of their potential influence on both respiratory impairment and health-related quality of life. Given the relatively small sample size and the exploratory nature of the study, the analyses were primarily based on univariate comparisons. No multivariable regression analysis was performed. Therefore, the possibility of residual confounding cannot be excluded.

Statistical analysis

Data were entered, organized, and analyzed using Microsoft Excel (Redmond, WA: Microsoft Corp.) and IBM SPSS Statistics version 26.0 (Armonk, NY: IBM Corp.). Categorical variables were expressed as frequencies and percentages, whereas continuous variables were summarized using means and standard deviations (SDs). Univariate analyses were performed to explore associations between sociodemographic, clinical, etiological, and treatment-related characteristics and the total K-BILD score. For comparisons between two independent groups, the independent-samples Student’s t-test was used when the assumptions of normality and homogeneity of variances were met; otherwise, the non-parametric Mann-Whitney U test was applied. For comparisons involving more than two independent groups, a one-way analysis of variance (ANOVA) was performed when the assumptions of normality and homogeneity of variances were satisfied. When these assumptions were not met, the non-parametric Kruskal-Wallis test was used. Given the relatively small sample size and the exploratory nature of the study, no multivariable regression analysis was performed. Therefore, the possibility of residual confounding cannot be excluded. All statistical tests were two-sided, and a p-value <0.05 was considered statistically significant.

## Results

Sociodemographic and epidemiological characteristics

Study Population

A total of 50 patients with fibrosing interstitial lung diseases (ILDs) were evaluated in the Department of Pulmonology at Avicenne Military Hospital in Marrakech, Morocco, during the seven-month study period from January to July 2026. All patients who attended the department during this period and fulfilled the predefined eligibility criteria were considered for inclusion. The final study population consisted of 50 patients with fibrosing ILDs confirmed by chest computed tomography (CT). The sociodemographic, clinical, etiological, and therapeutic characteristics of the participants were subsequently analyzed in relation to their health-related quality of life, as assessed using the French version of the K-BILD questionnaire.

Sex and Age Distribution

The study population consisted of 28 males (56%) and 22 females (44%), with a male-to-female ratio of 1.2 (Figure 1). Patients’ ages ranged from 50 to 86 years, with a mean age of 68 years. The 60-69-year age group was the most represented, comprising 23 patients (46%), followed by patients aged ≥70 years (21 patients, 42%) and 50-59 years (six patients, 12%) (Figure 2).

**Figure 1:**
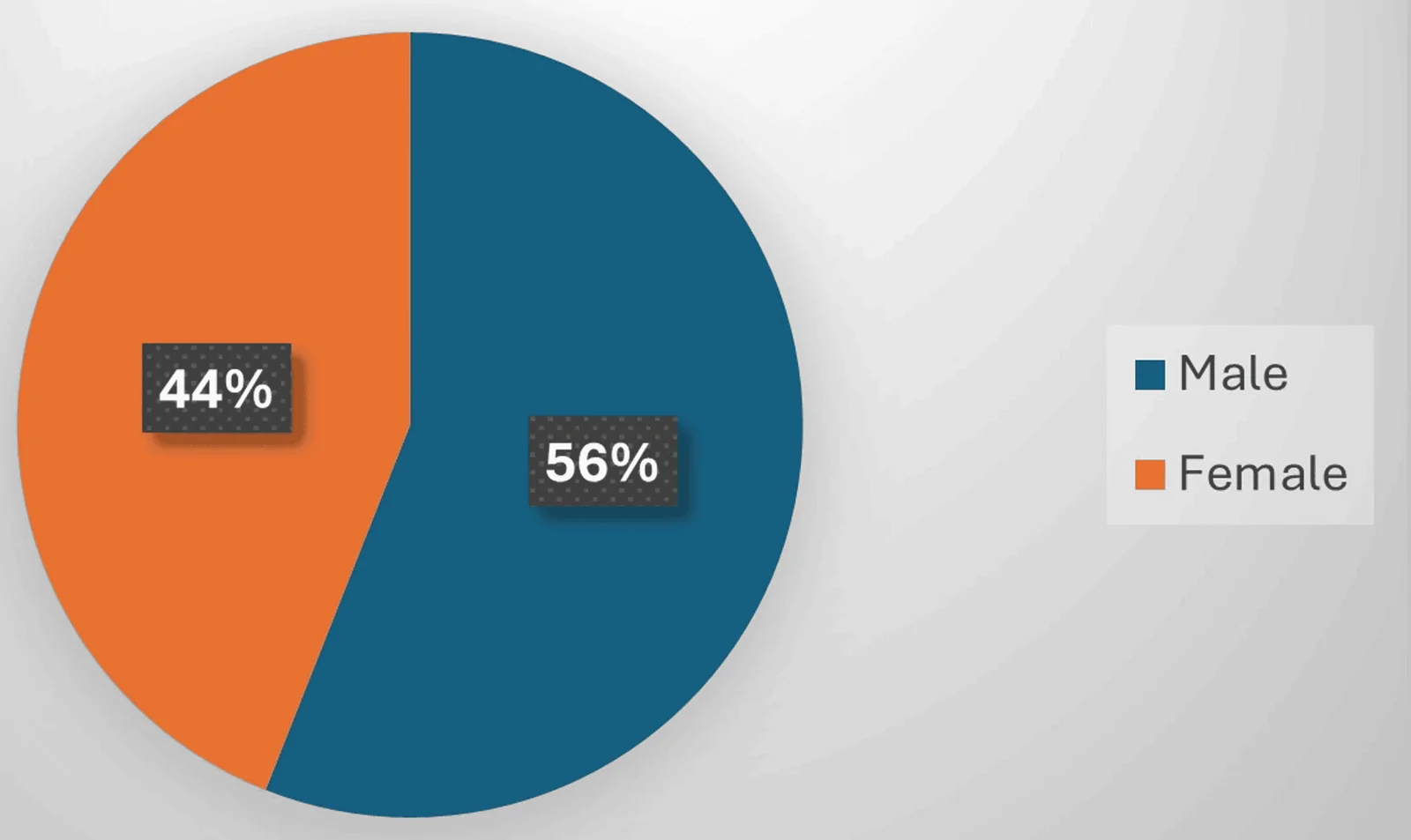
Distribution of patients according to sex.

**Figure 2:**
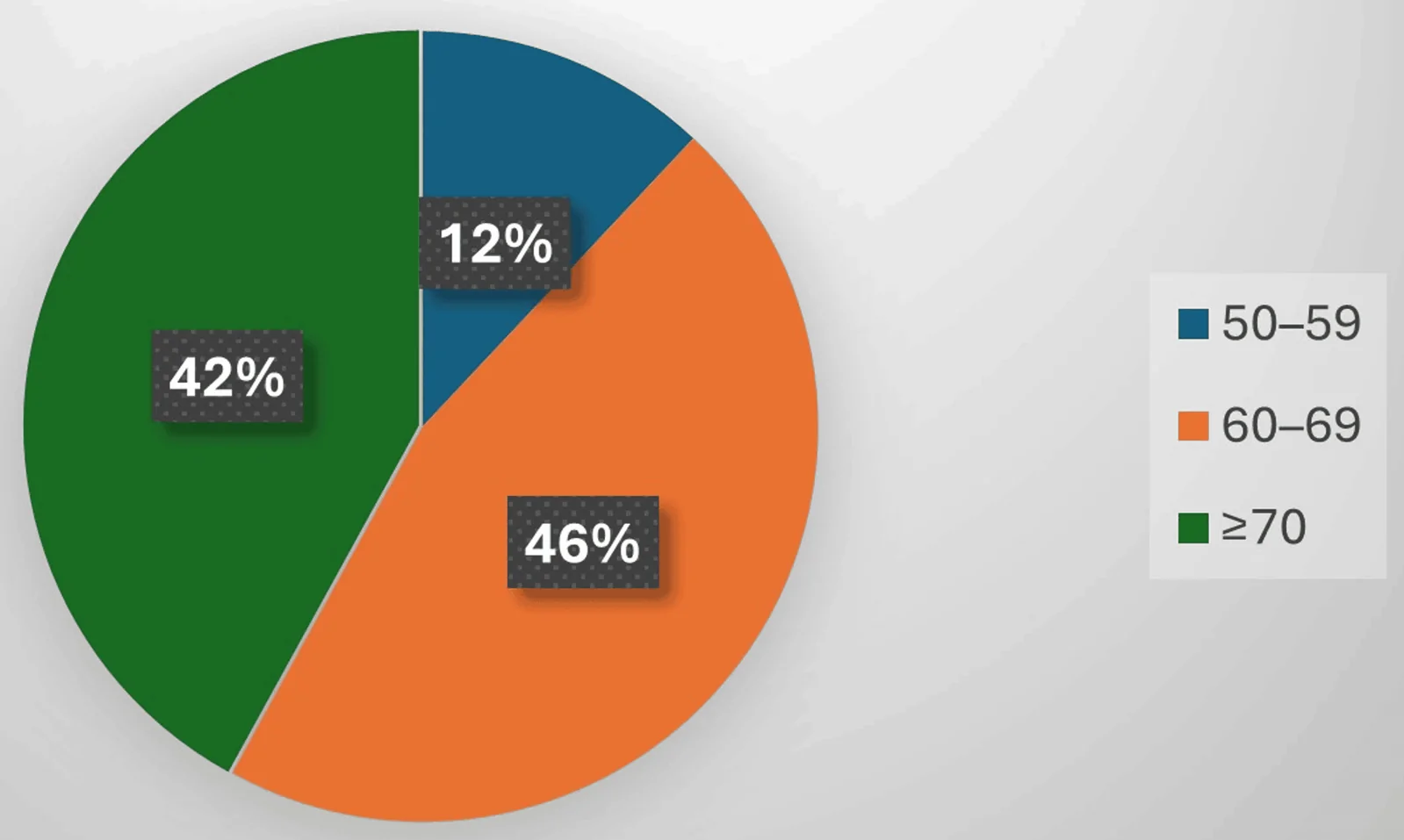
Distribution of patients according to age group.

Financial Independence

Among the study participants, 31 patients (62%) were financially independent, whereas 19 patients (38%) were financially dependent (Figure 3).

**Figure 3:**
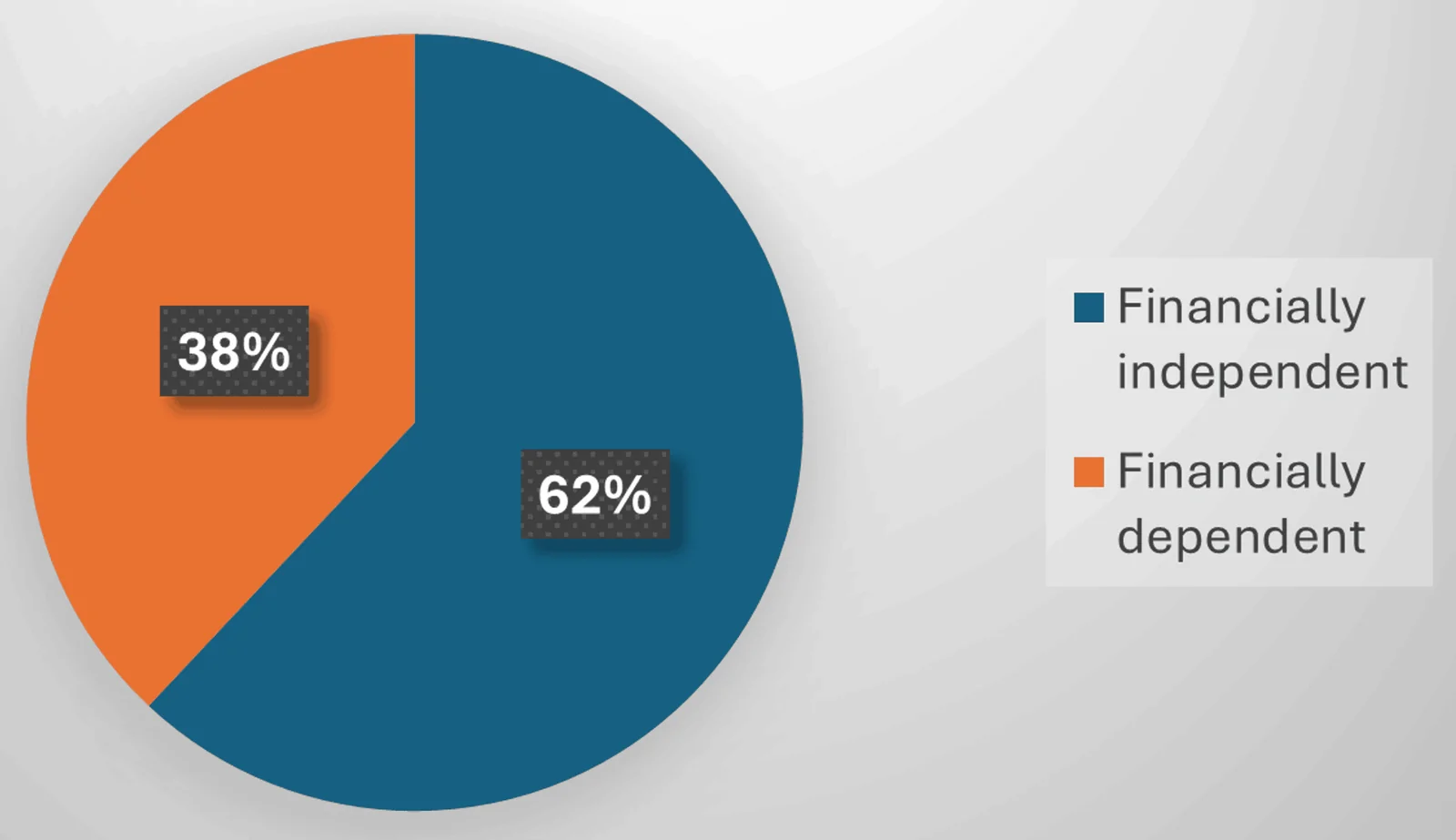
Distribution of patients according to financial independence.

Smoking History

Smoking status: In our study population, two patients (4%) were current smokers, 14 patients (28%) were former smokers, and 34 patients (68%) reported no tobacco exposure (Table 1).

**Table 1:** Distribution of patients according to smoking status.

Smoking status	Number of patients	Percentage
Current smoker	2	4
Ex-smoker	14	28
Never smoker	34	68
Total	50	100

Clinical characteristics

Disease History

Disease onset: Disease onset was most often progressive, occurring in 36 patients (72%), whereas acute onset (<3 weeks) occurred in 14 patients (28%) (Figure 4).

**Figure 4:**
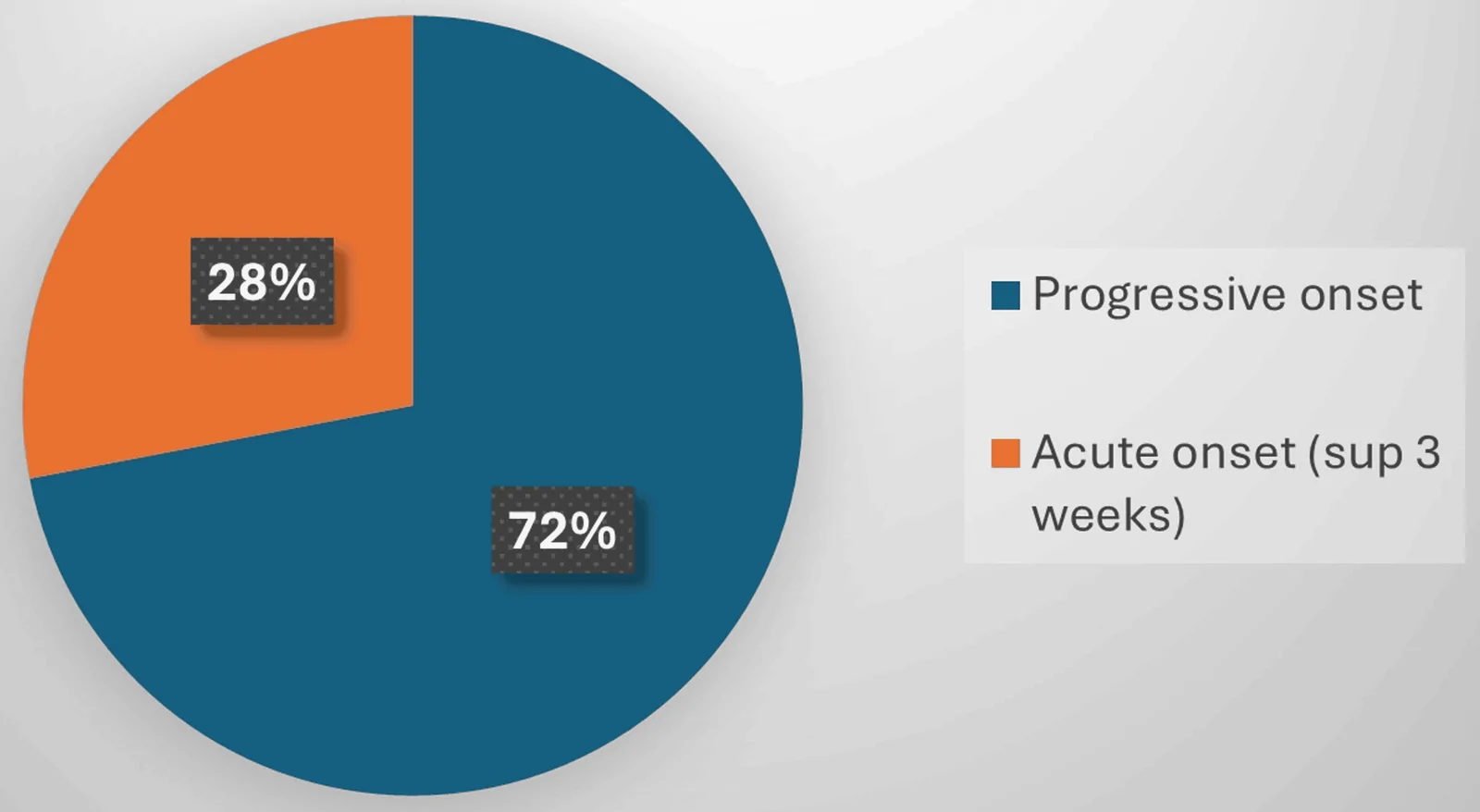
Distribution of patients according to disease onset.

Clinical Symptoms

Distribution of presenting symptoms: In our study population, dyspnea was the most frequently reported symptom, occurring in 47 patients (94%) and accounting for 50% of all reported symptoms, followed by cough, reported in 30 patients (60%) and accounting for 33% of all reported symptoms; percentages are not mutually exclusive, as individual patients may have presented with more than one symptom (Figure 5).

**Figure 5:**
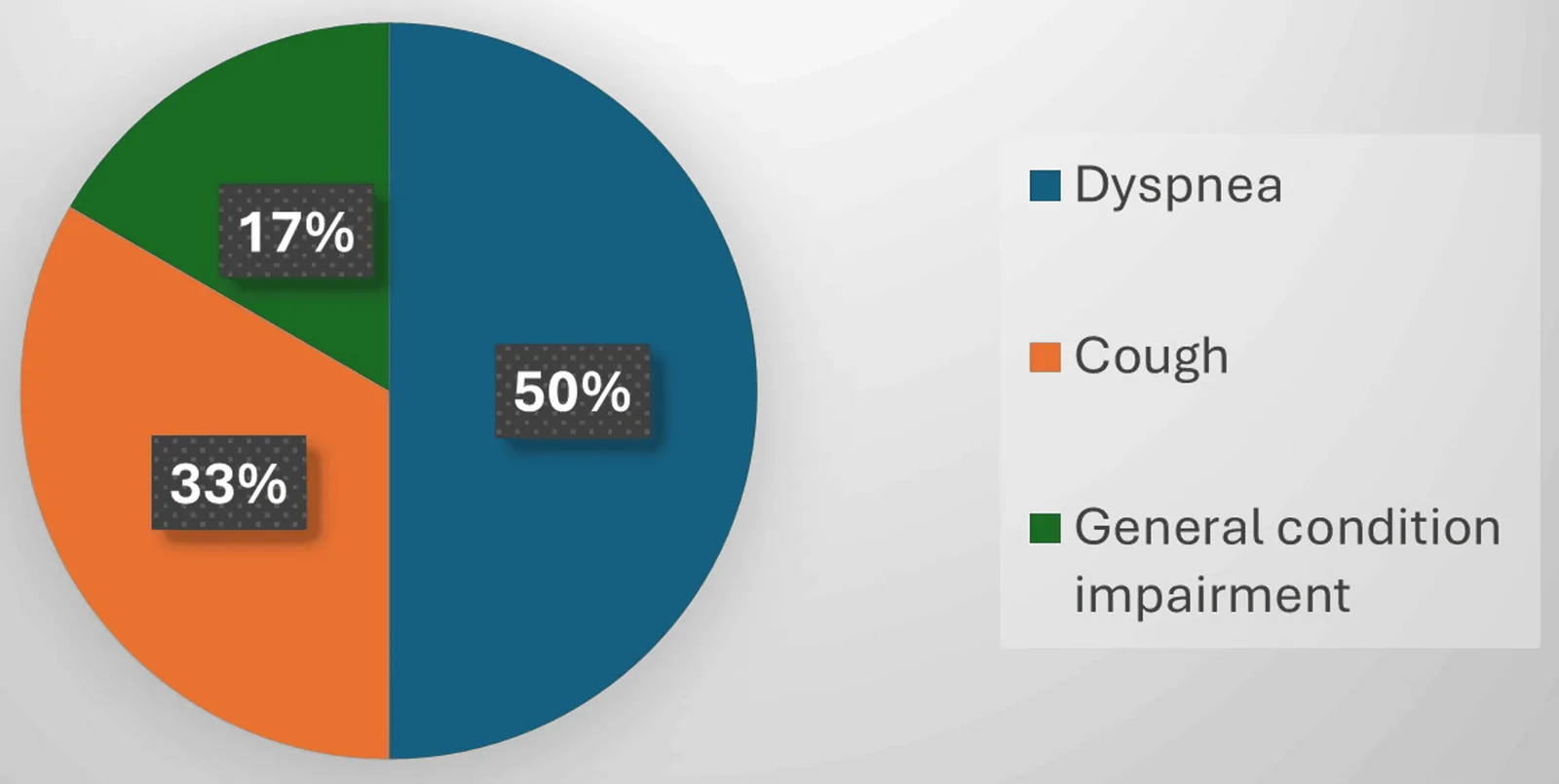
Distribution of respiratory symptoms among the study population.

Dyspnea severity: Dyspnea was reported in 47 patients (94%). According to the modified Medical Research Council (mMRC) dyspnea scale, dyspnea was classified as grade 1 in three patients (6%), grade 2 in 17 patients (34%), grade 3 in 19 patients (38%), and grade 4 in eight patients (16%) (Table 2).

**Table 2:** Distribution of patients according to dyspnea severity based on the mMRC scale. mMRC: modified Medical Research Council

Variable	Stage of mMRC	Number of patients	Percentage
Dyspnea	1	3	6
	2	17	34
	3	19	38
	4	8	16
Total		47	94

Clinical Examination

Resting room-air oxygen saturation: In our study population, mean resting oxygen saturation on room air was 90%, with values ranging from 80% to 100%. The 90-94% range was the most frequently observed, accounting for 22 patients (44%) (Table 3).

**Table 3:** Distribution of patients according to resting SpO₂ on room air.

SpO_2_	Frequency (n)	Percentage
95-100%	21	42
90-94%	22	44
80-89%	7	14
Total	50	100

Pulmonary auscultation: On pulmonary auscultation, 45 patients (90%) had bilateral dry fine inspiratory crackles with a Velcro-like quality, whereas five patients (10%) had no crackles (Figure 6).

**Figure 6:**
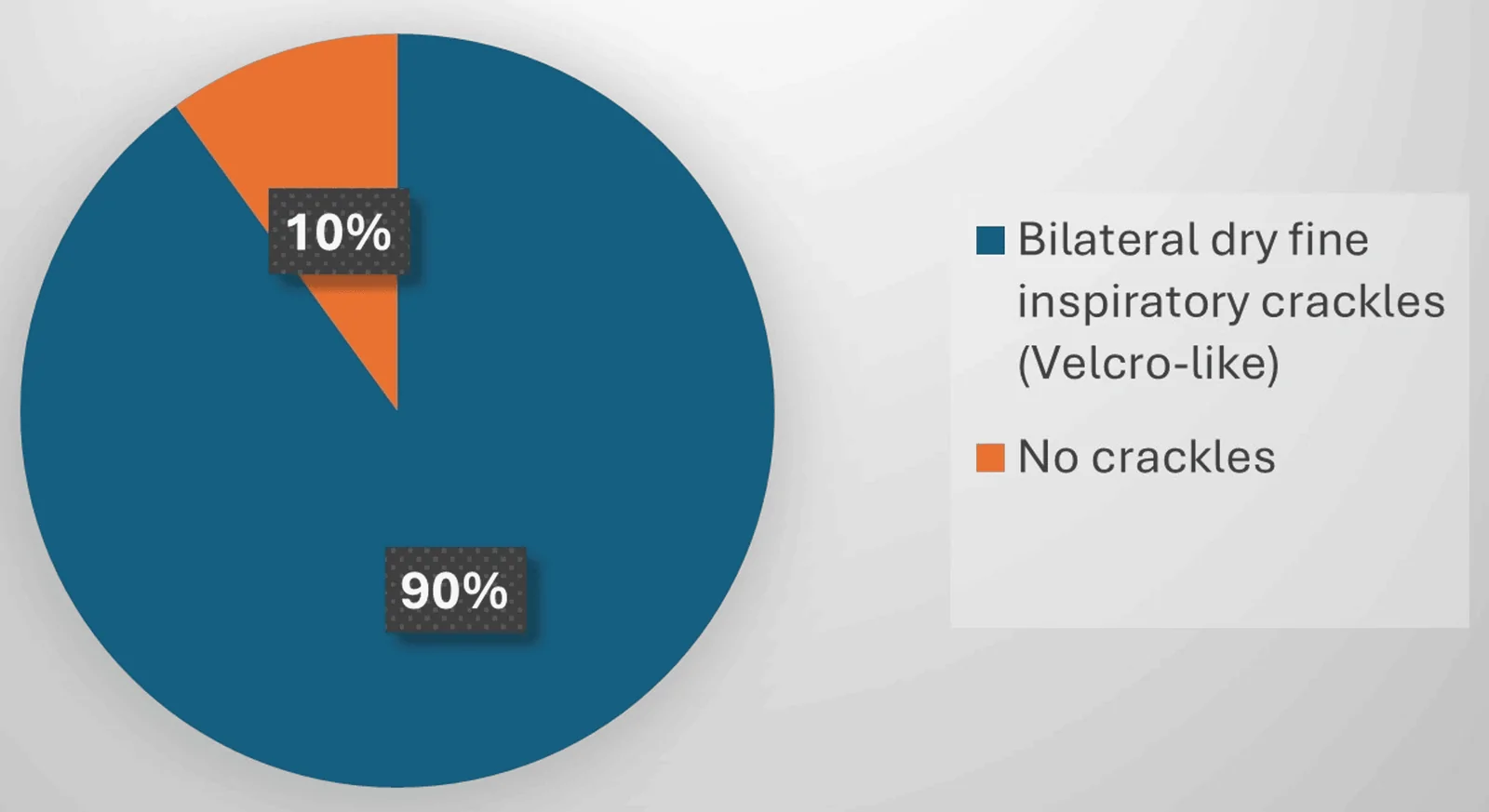
Distribution of patients according to pulmonary auscultation findings.

Etiological characteristics: In our study population, idiopathic fibrosing interstitial lung diseases (ILDs) constituted the most frequent etiological category, occurring in 29 patients (58%), followed by hypersensitivity pneumonitis in 12 patients (24%), non-specific interstitial pneumonia (NSIP) in seven patients (14%), sarcoidosis in one patient (2%), and systemic sclerosis in one patient (2%) (Figure 7).

**Figure 7:**
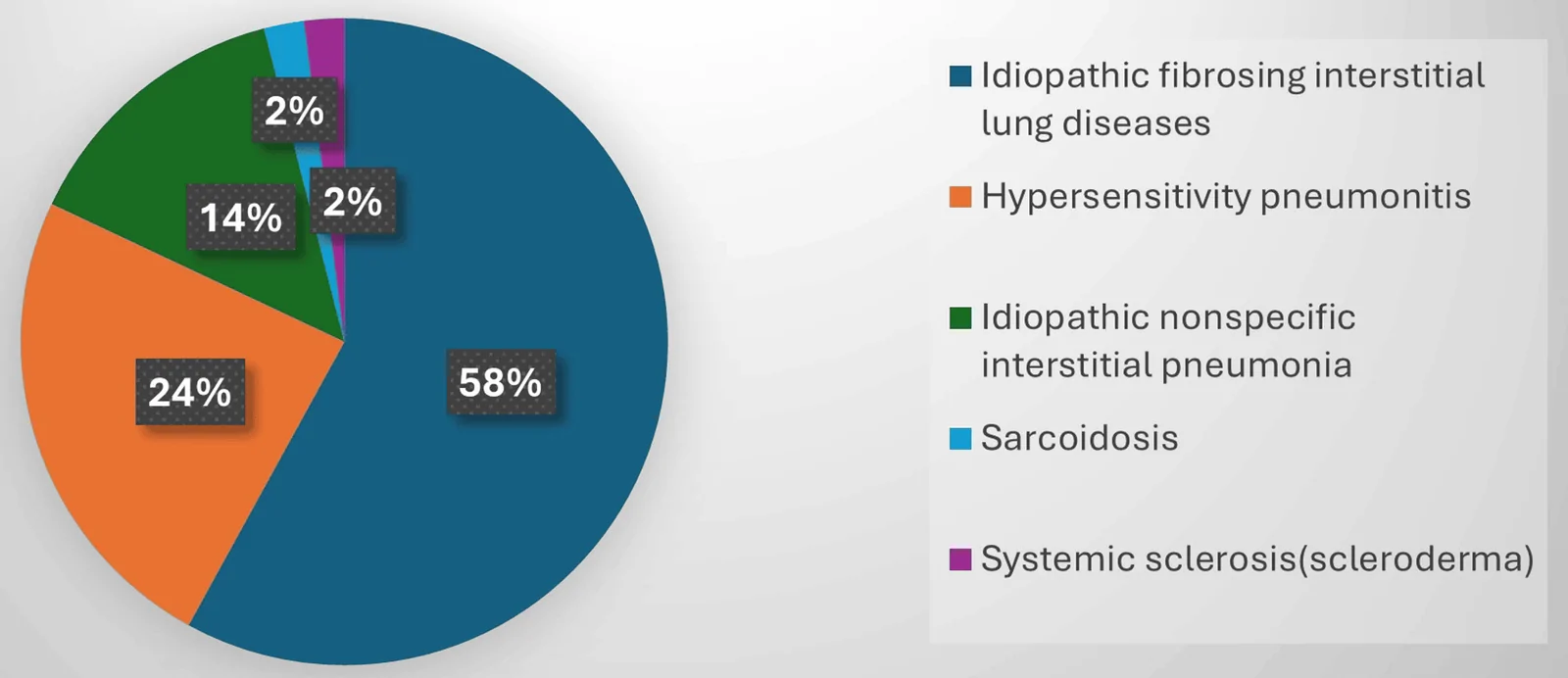
Etiological distribution of fibrosing interstitial lung diseases.

Therapeutic characteristics

Treatment Modalities

Etiological treatment: In our study population, long-term corticosteroid therapy was the most commonly used treatment, prescribed in 25 patients (50%), whereas antifibrotic therapy, particularly nintedanib, was used in only three patients (6%) (Table 4).

**Table 4:** Distribution of patients according to etiological treatment.

Treatment	Number of patients	Percentage
Long-term corticosteroid therapy	25	50
Azathioprine	4	8
Nintedanib	3	6
No treatment	18	36

Health-Related Quality of Life

The mean total K-BILD score was 48.43±19.77, indicating an overall impairment in health status in the study population. The breathlessness and activities domain had the lowest mean score (32.83±27.32), whereas higher mean scores were observed for the psychological domain (53.90±18.65) and the chest symptoms domain (56.44±22.76). Item-level analysis showed that item 1, which assesses breathlessness, had the lowest mean score (23.66±1.62), indicating greater impairment in this aspect of health status, as lower K-BILD scores reflect poorer health status (Table 5).

**Table 5:** Distribution of total and domain-specific K-BILD scores. K-BILD: King’s Brief Interstitial Lung Disease; HRQoL: health-related quality of life

K-BILD measure	Mean±SD	Interpretation
Total K-BILD score	48.43±19.77	Moderately impaired HRQoL
Breathlessness and activities domain	32.83±27.32	Lower domain score
Psychological domain	53.90±18.65	Intermediate domain score
Chest symptoms domain	56.44±22.76	Highest domain score
Item 1 - breathlessness	23.66±1.62	Low score, indicating greater symptom burden

Financial impact of the disease: Regarding the financial impact of the disease, 21 patients (42%) reported frequent financial difficulties, while nine patients (18%) reported occasional difficulties and six patients (12%) reported rare difficulties. In contrast, five patients (10%) indicated that they had never experienced financial difficulties related to their lung disease. At the two extremes of the response scale, four patients (8%) reported experiencing financial difficulties almost always, whereas one patient (2%) reported experiencing them constantly, highlighting the presence of a particularly vulnerable subgroup. Finally, four additional patients (8%) could not be classified (Figure 8).

**Figure 8:**
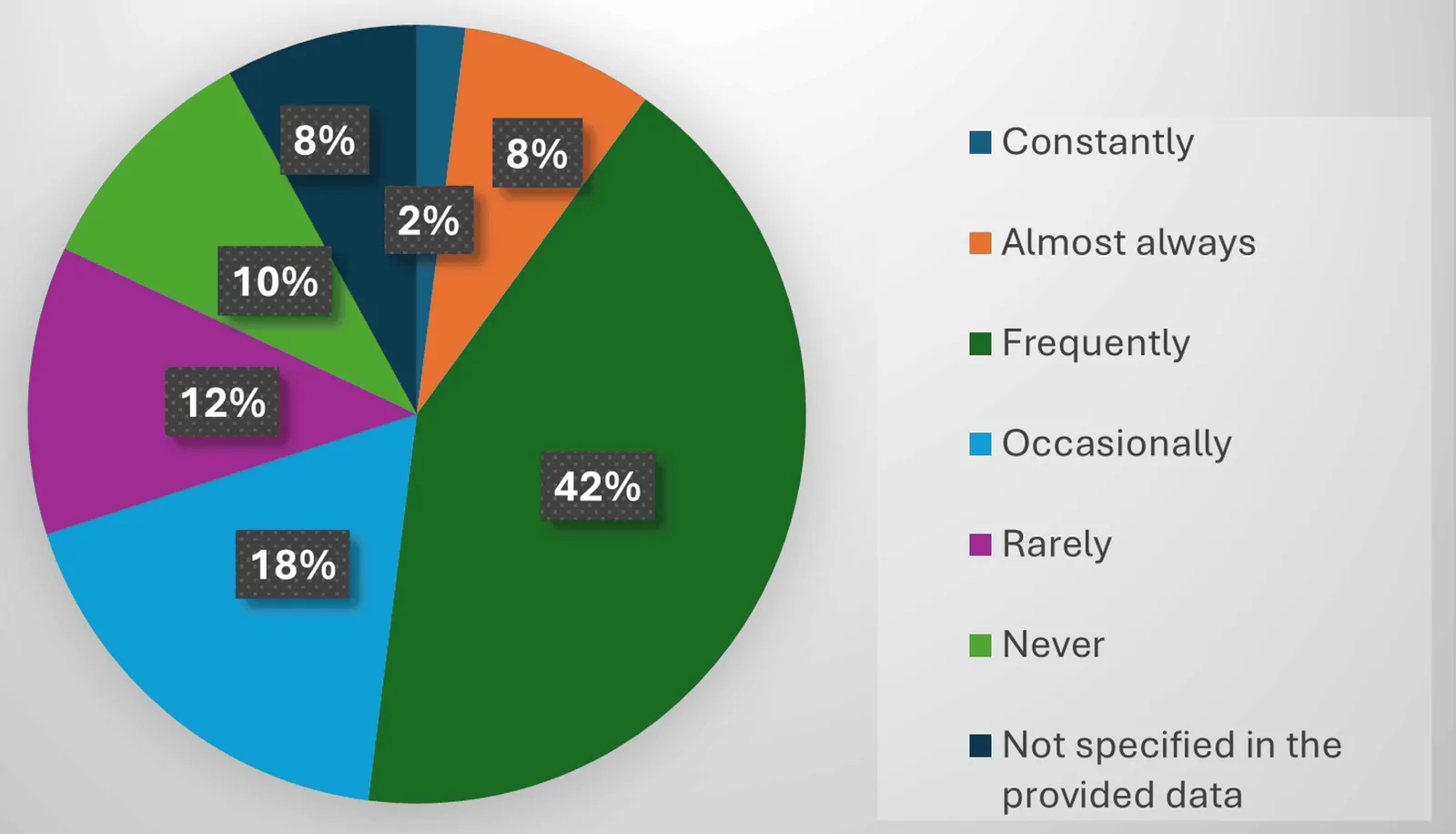
Financial impact of interstitial lung disease on patients.

Comparative Analysis

Association between sociodemographic and treatment-related characteristics and the mean total K-BILD score: Univariate analysis of the associations between sociodemographic and treatment-related characteristics and the mean total K-BILD score showed no statistically significant associations. This may be partly explained by the relatively small sample size and the absence of multivariable analysis.

Association between the mean total K-BILD score and resting room-air oxygen saturation: A one-way ANOVA demonstrated a statistically significant difference in mean total K-BILD scores across the three resting room-air SpO₂ groups (F {2, 47}=5.91, p=0.005) (Table 6).

**Table 6:** Comparison of the mean total quality of life (QoL) score according to SpO₂.

SPO_2 _	n	Mean±SD	Statistical test	F	p-Value
95-100%	21	58.33±15.24	One-way ANOVA	5.91	0.005
90-94%	22	42.96±20.84			
80-89%	7	35.88±15.38			

## Discussion

In the present study, a total of 50 patients with fibrosing interstitial lung diseases (ILDs) were included, with a slight male predominance - 56% (28 patients) were men and 44% (22 patients) were women, corresponding to a male-to-female ratio of 1.2. This distribution differs from that reported by Achour et al., who included 33 patients, of whom 29 (88%) were women and four (12%) were men, with a male-to-female ratio of 0.13 [11]. This difference may be explained by the fact that their study was conducted in an internal medicine department and included a relatively small sample compared with our study. The mean age in our study was 68 years (range: 50-86 years), with the 60-69-year age group being the most represented. This finding is consistent with that reported by Jellam et al., who reported a mean age of 61.5 years (range: 36-78 years) [12]. In the present study, disease onset was predominantly progressive, occurring in 72% of patients. The most common symptom was dyspnea, reported by 47 patients (94%), followed by cough in 30 patients (60%). These findings are consistent with those reported by Knaz et al., in whose study exertional dyspnea was present in 92% of patients, followed by dry cough in 67% and extrapulmonary manifestations in 24% of cases [13]. In the present cohort, 45 patients (90%) presented with bilateral fine inspiratory crackles. This proportion was higher than that reported by Ijim et al., who found fine inspiratory crackles in 21 patients (46.66%) [8]. This difference may be related to differences in sample size and patient characteristics. In the present study, the etiological profile was dominated by idiopathic fibrosing ILDs, which were identified in 29 patients (58%). This proportion was higher than that reported by Ijim et al. at Mohammed VI University Hospital in Marrakech, where the etiology of chronic fibrosing ILD was established in 32 patients (71.7%), including 15 cases (33.3%) of secondary ILD and 17 cases (37.8%) of idiopathic ILD, while the etiology remained undetermined in 13 patients (28.9%) [8]. Conversely, our findings were relatively similar to those reported by Villeneuve et al. in Haute-Garonne, France, where idiopathic ILDs accounted for 55.8% of fibrosing ILDs [6]. Among secondary fibrosing ILDs, hypersensitivity pneumonitis was the most frequent etiology in our cohort, affecting 12 patients (24%). In contrast, in the study by Nokra et al. conducted at Errazi University Hospital between January 2020 and August 2022, the etiological assessment showed that sarcoidosis was the predominant cause of secondary fibrosing ILD, accounting for 22% of cases [14]. In the present study, long-term corticosteroid therapy was the most frequently used treatment. This finding is consistent with that reported by Nokra et al. at Errazi University Hospital, where corticosteroid therapy was prescribed in 66.6% of patients, immunosuppressive therapy in 13.7%, and antifibrotic therapy in 11.7% [14]. Long-term oxygen therapy was prescribed in 15.7% of patients [14]. In our study, the mean total K-BILD score was 48.43±19.77, indicating a moderate impairment in health-related quality of life among patients with fibrosing ILD. This score was slightly lower than that reported by Jeanbat et al. in 2020 in a French university hospital, where the mean K-BILD score was 58.4±12.7 [15]. Item-level analysis showed that item 1, assessing breathlessness, was the most impaired, with a mean score of 23.66±1.62, indicating substantial impairment in this aspect of health status among our patients. In contrast, higher scores were observed in the psychological domain (53.90±18.65) and the chest symptoms domain (56.44±22.76), suggesting that these dimensions were relatively better preserved in our study population. These findings are consistent with those reported by Szentes et al., who investigated the correlations between K-BILD and EQ-5D dimensions [16]. The strongest correlation was observed between the breathlessness and activities domain of the K-BILD and the usual activities dimension of the EQ-5D [16]. This finding highlights the important contribution of dyspnea and activity limitation to overall health status and quality of life in patients with ILD. When comparing the mean total K-BILD score according to oxygen saturation, we observed a statistically significant association between resting oxygen saturation on room air and health-related quality of life (p=0.005). This finding is consistent with the pathophysiological characteristics of ILDs, which are frequently associated with impaired gas exchange and hypoxemia, particularly during exertion. In the study by Achour et al., physical activity was limited by dyspnea in 41% of patients and was associated with an impact on daily activities in 15% of cases [11]. Similarly, in the study by Birring et al., strong associations were reported between K-BILD scores and relevant clinical measures in patients with ILD [17]. Among stable patients without changes in global health status (n = 187), the reported correlation coefficients were 0.74 for the total K-BILD score, 0.72 for the breathlessness and activities domain, 0.67 for the psychological domain, and 0.71 for the chest symptoms domain [17]. These findings further support the relevance of K-BILD as a patient-reported measure of health status in patients with ILD. Another study conducted at Souss-Massa University Hospital in Agadir between 2022 and 2024 evaluated health-related quality of life using the Short Form-36 Health Survey (SF-36) and World Health Organization Quality of Life-100 (WHOQOL-100) questionnaires, in contrast to our study, which used the K-BILD questionnaire. The authors reported impaired health-related quality of life associated with higher mMRC dyspnea scores. During follow-up, changes in forced vital capacity (FVC), diffusing capacity of the lung for carbon monoxide (DLCO), and mMRC dyspnea grade were associated with changes in health-related quality of life [2]. These findings are consistent with our results and further highlight the relationship between respiratory impairment, dyspnea, and patient-reported quality of life. Our results are consistent with those reported by Rautajoki et al. in their study assessing health-related quality of life using the K-BILD questionnaire [18]. The total K-BILD score remained higher among patients with a baseline mMRC score of 0-1 than among those with an mMRC score of 2-4, and the total score decreased only modestly in both groups, with median declines of 3.3 and 4.8 points among patients with mMRC scores of 0-1 and 2-4, respectively [18]. This observation further supports the important contribution of dyspnea and functional limitation to impaired health-related quality of life in patients with fibrosing ILD.

Limitations

This study has several limitations. First, its single-center design, involving only 50 patients recruited from a single department, may limit the generalizability of our findings to other healthcare settings and patient populations. Second, the absence of longitudinal follow-up prevented us from assessing changes in K-BILD scores over time. In addition, the lack of pulmonary function and exercise capacity data, including spirometry, body plethysmography, diffusing capacity for carbon monoxide (DLCO), and the six-min walk test, represents an important limitation, as we were unable to assess the relationship between health-related quality of life and pulmonary functional parameters or exercise capacity. The absence of multivariable analysis also represents a limitation, particularly given the relatively small sample size. Therefore, the associations observed in our study cannot be interpreted as independent associations after adjustment for potential confounding factors. Furthermore, effect-size measures were not calculated, limiting the assessment of the magnitude and clinical relevance of the observed associations. Finally, although the K-BILD is a validated and disease-specific instrument for interstitial lung diseases, it relies on patient-reported outcomes. Its scores may therefore be influenced by individual perceptions of symptoms and psychological factors. Given these limitations, particularly the single-center design, limited sample size, and absence of longitudinal follow-up and multivariable analysis, our findings should be interpreted with caution and cannot be directly extrapolated to other patient populations.

## Conclusions

In our population of patients with fibrosing interstitial lung diseases (ILDs), health-related quality of life was overall impaired, with a mean total K-BILD score of 48.43±19.77. A one-way ANOVA demonstrated a statistically significant difference in mean total K-BILD scores across the three groups of resting room-air SpO_2_ (F {2,47}=5.91; p=0.005). These findings highlight the potential value of systematic assessment of health-related quality of life as part of the comprehensive management of patients with fibrosing ILDs. Further multicenter longitudinal studies involving larger patient populations and incorporating pulmonary function testing are warranted to confirm these associations.

## Appendices

**Table 7:** Domains, item numbers, content assessed, and corresponding questions. Each item is rated on a seven-point Likert scale: always (1), almost always (2), several times (3), sometimes (4), occasionally (5), rarely (6), never (7). The questionnaire comprises three domains: breathlessness and activities (items 1, 4, 11, and 13), psychological (items 3, 5, 6, 8, 10, 12, and 14), and chest symptoms (items 2, 7, and 9); item 15 assesses the financial impact of the disease.

Domain	Item number	Content assessed/item theme	Questions
Breathlessness and activities	1	Impact of breathlessness on physical activities	During the past two weeks, have you been short of breath when climbing stairs or walking up a slope or hill?
	4	Breathlessness-related limitation in daily activities	During the past two weeks, have you avoided doing certain activities that make you short of breath?
	11	Impact of breathlessness on activities	During the past two weeks, has your lung disease affected your work or other daily activities?
	13	Ability to perform certain activities despite respiratory disease	During the past two weeks, to what extent has your lung disease prevented you from carrying objects, such as groceries?
Psychological	3	Concern or worry related to respiratory disease	During the past two weeks, have you been worried about the severity of your lung symptoms?
	5	Sense of control or mastery over the disease	During the past two weeks, have you felt in control of your lung disease?
	6	Emotional impact of the disease	During the past two weeks, have your lung symptoms made you upset or depressed?
	8	Concern about health status	During the past two weeks, how often have you felt that your lung disease was getting worse?
	10	Feelings of discouragement or frustration	During the past two weeks, have your usual lung symptoms become worse?
	12	Psychological impact of the disease on daily life	During the past two weeks, has your lung disease made you feel anxious?
	14	Feelings of anxiety or worry related to symptoms	During the past two weeks, has your lung disease made you think more about the end of your life?
Chest symptoms	2	Presence and impact of respiratory/chest symptoms	During the past two weeks, because of your lung problem, have you experienced a feeling of tightness or pressure in your chest?
	7	Intensity or frequency of chest symptoms	During the past two weeks, how often have you experienced wheezing in your chest?
	9	Impact of cough or other respiratory symptoms	During the past two weeks, have you felt the need to breathe deeply and frequently, also referred to as a “hunger for air” sensation?
Financial impact	15	Financial impact of the disease	During the past two weeks, has your financial situation worsened because of your lung disease?
